# The Hypophosphites

**Published:** 1858-07

**Authors:** James R. Nichols

**Affiliations:** Boston


*The Hypophosphites.* By James R. Nichols, M. D., Boston.

[Communicated for the Boston Medical and Surgical Journal.]

The importance of the element phosphorus in the human economy has not been fully appreciated until within quite a recent period. The amount present in the brain, as shown by investigations at the Cambridge laboratory, a few years since, is much larger than was supposed. Not only had the best chemists of Europe fallen into error in their estimations of the quantity of phosphorus, bus also in that of sulphur, the element so closely allied to phosphorus in its uses and chemical affinities.

The vital importance of these agents in maintaining a normal condition of the system can be understood by a consideration of the probable fact, that in all the operations of the mind, in every effort requiring an expenditure of nervous force, they are called into action. In their rapid oxidation in the brain, on occasions of great intellectual effort, there may be a nearer approximation to literal truth in the remark, that there are "thoughts that *burn,*" than is generally supposed.

Not only is chemical science capable of pointing out the exact chemical constitution of the body, and the changes and transformations which are constantly occurring, but it has proved competent to direct us respecting the proper methods by which certain elements or agents may be furnished when pathological symptoms indicate an insufficient supply.

The use of that class of salts known as hypophosphites, offers the most direct and philosophical means of supplying phosphorus to the system. The small amount of oxygen in combination with this element in the hypophosphorous acid which unites with the alkaline carbonates, the bases of these salts, is favorable to easy decomposition in the economy. By the changes which result from further oxidation, nascent phosphorus and phosphates are liberated. The phosphorus thus set free is certainly in a condition most favorable to the fulfilment of its design and high office in the brain and nervous system. Whether the phosphates, as such, or by further change, are capable of exerting specific and desirable influence, must be regarded as a matter of some doubt, although they have been administered in the form of "syrups of the phosphates" for a considerable time, by distinguished physicians.

When the oxide of phosphorus is placed in contact with hot milk of lime, hypophosphite, phosphate and carbonate of lime, hydrogen and phosphuretted hydrogen result from the chemical changes which occur. The hypophosphite of lime is soluble, and remains in solution while the insoluble phosphate and carbonate are suspended, or fall to the bottom of the vessel as a precipitate. In the preparation of the hypophosphites, the usual method has been to boil the phosphorus of commerce in milk of lime until the formation of phosphuretted hydrogen ceases, then filtering the liquid, working the precipitate, and after concentrating the clear solution, allowing it to crystallize into a dry salt. Mr. Shaffer, of Louisville, proposes, in the last number of the *Journal of Pharmacy*, to first oxidize the phosphorus by a current of air thrown upon it, while fused under hot water, and then to place it in milk of lime, as in the former process, for the formation of the hypophosphite.

This process I have found tedious, and perhaps not less objectionable than the other. The product is not essentially larger. It may be much facilitated by employing a current of mingled air and steam in preparing the oxide of phosphorus, instead of the air current. The white vapors of phosphoric acid produced are very dense and abundant, and the process should be conducted under a strong draught. As an experiment, the passing of an air current upon melted phosphorus in water, is beautiful. The intensely vivid flame and streams of light, commingled with sparks, beneath the water, afford a pleasing spectacle, when the operation is conducted upon a large scale.

The alkaline hypophosphites of soda, potassa and ammonia are readily obtained by reaction with the lime salts. The iron salt may be obtained by precipitating a solution of hypophosphite of soda with one of sesquisulphate of iron.

As a source from which to supply phosphorus to the system, hypophosphorous acid must be regarded as of the highest importance. What phosphoric acid may have failed to do, in supplying the waste of phosphorus, it is almost certain that the acid containing the less amount of oxygen is capable of accomplishing. Uncombined with the alkalies, it should receive attention as a remedial agent.

It is undoubtedly the experience of medical gentlemen that most new chemical remedies fail to accomplish the expectations of the introducer, and therefore much hesitation may be expected in making trial of the hypophosphites, and the acid from which they result. Dr. Churchill, to whom we are indebted for calling attention to them, is confident that "they will occupy one of the most conspicuous places in the materia medica." "The effect of these salts," he says, "upon the tuberculous diathesis is immediate, the general symptoms of the disease disappearing with a rapidity which is really marvellous."

Although specifically prescribed for phthisis, by Dr. Churchill, it is evident their beneficial effects are not limited to this one variety of disease. They would seem to be most appropriate remedies in a large class of affections resulting from loss of nervous force, and perhaps in cases of mental aberration not resulting from family idiosyncracies; also in many of the diseases of infancy, where there is want of vital action, and where the osseous system is defective. A clear understanding of the chemical nature and characteristics of the remedies under discussion, will enable physicians to apply them in a large class of diseases where their use seems to be indicated.

The dose of these salts of lime, soda, potassa and ammonia, is about ten grains to adults, given two or three times in the twenty-four hours.—— They may be combined in the form of syrup, or with lactose (sugar or milk,) or glycerine. All the salts are more or less deliquescent, and therefore it is important they should be placed under the protection of some agent capable of preserving them from change.

